# Needs assessment of basic gastrointestinal endoscopy training: A qualitative study in Indonesia

**DOI:** 10.1002/jgh3.13004

**Published:** 2023-11-20

**Authors:** Hasan Maulahela, Nagita G Annisa, Marcellus Simadibrata, Ari F Syam, Ardi Findyartini, Wresti Indriatmi, Roy Soetikno

**Affiliations:** ^1^ Doctoral Program in Medicine Sciences Faculty of Medicine Universitas Indonesia, Division of Gastroenterology, Pancreatobilliary and Digestive Endoscopy, Cipto Mangunkusumo General Central Hospital Universitas Indonesia Jakarta Indonesia; ^2^ Faculty of Medicine Universitas Indonesia Dr Cipto Mangunkusumo General Central National Hospital Jakarta Indonesia; ^3^ Department of Medical Education, Faculty of Medicine Universitas Indonesia Dr Cipto Mangunkusumo General Central National Hospital Jakarta Indonesia; ^4^ Department of Dermatology and Venereology, Faculty of Medicine Universitas Indonesia Dr Cipto Mangunkusumo General Central National Hospital Jakarta Indonesia

**Keywords:** gastrointestinal endoscopy, medical education, medical training

## Abstract

**Background and Aim:**

Gastointestinal endoscopy is a complex practical skill, and training and experience are required to ensure the accuracy and safety of the procedures. Therefore, proper endoscopy training is needed to provide highly skilled endoscopists. This study explores the learning experience and assesses the need for endoscopy training in Indonesia from an endoscopy trainee's point of view. Limitations from the current training model and the trainees' suggestions hopefully will become a foundation for the future endoscopy training model in Indonesia.

**Methods:**

A total of 132 current endoscopy trainees and graduates of endoscopy training from various centers in Indonesia completed an online qualitative survey regarding their endoscopy training experience, their satisfaction with the current training method, barriers to achieving competency, and their suggestions for future training. Data were subjected to descriptive and qualitative analysis using content analysis.

**Results:**

We found variations in the trainee's learning experience regarding the training supervision, feedback, and assessment methods. The most common endoscopy training methods were observation and direct practice with supervision. There was only a low proportion of simulator use (25%). The most found concept in barriers to achieving competency was “insufficient number of patients.” Meanwhile, the most found concept in suggestions for future training methods was “increasing the variety of cases and procedures.”

**Conclusion:**

Our findings suggest that there are still variations in endoscopy training methods in Indonesia. Therefore, we propose to design a standardized endoscopy training program to ensure the competence of endoscopy trainees and better care for endoscopic patients. Simulators might be used to increase the trainees' competence in settings with low numbers of patients or cases.

## Graphical abstract

Needs assessment of basic gastrointestinal endoscopy training based on a national survey from endoscopy trainees and graduates in Indonesia.

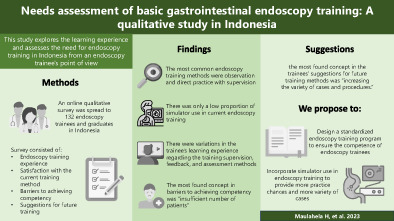

## Introduction

Indonesia is one of the world's most populous countries, with an estimated population of 273.8 million in 2021. Among the population, gastrointestinal (GI) diseases are one of Indonesia's most common health problems. In 2010, diarrhea, gastroenteritis, and colitis ranked fifth as the most frequent disease in the outpatient clinic in Indonesian hospitals, while dyspepsia ranked sixth. Diarrhea and gastroenteritis are among the most frequent diseases in the inpatient ward in Indonesian hospitals. GI disease is also ranked fifth as the highest cause of mortality in Indonesia (case fatality rate [CFR] 2.91%). GI endoscopy is an essential procedure in the diagnosis and management of various GI diseases such as gastric ulcers, polyps, GI bleeding, and GI cancers.^1^

Considering the numerous roles of endoscopy in the management of GI diseases, it is crucial for endoscopists to perform the procedure well. GI endoscopy is a complex practical skill, and training and experience are required to ensure the accuracy and safety of the procedures. Therefore, proper endoscopy training is needed to provide highly skilled endoscopists. Based on 2013 data, there were 516 GI endoscopists in Indonesia, consisting of internists, pediatricians, surgeons, and general practitioners. However, until 2012 there were only 10 training centers in Indonesia, with 7 of them located on Java Island and the other 3 in Sumatra, Bali, and Sulawesi Islands. This number is very low, considering the size of the Indonesian population and the large number of gastrointestinal diseases.^2^

Traditionally, endoscopy training is performed through an apprenticeship model. However, this model has several limitations, including varied results between the trainees due to limited and varied patient and case exposure.^3^ Currently, there is no standardized method for endoscopy training in Indonesia. There is also very little research concerning endoscopy training methods. Therefore, this qualitative study is conducted to explore the learning experience and assess the need for endoscopy training in Indonesia from the endoscopy trainee's point of view. We hope that understanding the current training method's needs and limitations could become a foundation for designing a better, more standardized endoscopy training model in Indonesia.

## Methods

### Data collection

A qualitative study was chosen to explore the endoscopy trainees' personal views and learning experiences. Data were collected by survey using an online questionnaire from February 2023 to June 2023. The online questionnaire was spread through emails to all members of The Indonesian Society for Gastrointestinal Endoscopy, which consists of 537 gastroenterology subspecialists and practicing GI endoscopists in Indonesia. The questionnaire consisted of close‐ and open‐ended questions regarding personal information (e.g. gender, age, location, etc.), teaching methods used in endoscopy training, use of simulators in endoscopy training, satisfaction with the abilities/competencies achieved, and barriers to teaching methods used in achieving competency (Appendix S1, Supporting information). The questionnaire was written in Indonesian and validated by an expert panel before distribution. Ethical clearance was given by the Health Research Ethics Committee, Faculty of Medicine, Universitas Indonesia‐Cipto Mangunkusumo General Hospital (protocol no. 22‐11‐1302 dated 7 November 2022).

### Participants

Participants of this study were current trainees or graduates of endoscopy training from various endoscopy training centers in Indonesia. There was no limitation in participants' background or training year. Participants included internal medicine specialists, gastroenterology fellows, or gastroenterology subspecialists who had undertaken endoscopy training in any training center from any year who were willing to fill out the questionnaire and consented to participate in the study.

### Data analysis

Our questionnaire consisted of close‐ and open‐ended questions. Data from close‐ended questions were analyzed with descriptive analysis and would be presented in the form of frequency tables and bar or pie charts. Data from open‐ended questions included participants' views on barriers to achieving competency in endoscopy training and their suggestions to improve future endoscopy training. These data were analyzed qualitatively with content analysis. Boettger and Palmer defined content analysis as “a research technique for making replicable and valid inferences from texts (and other meaningful matter) in the contexts of their use.”^4^ The authors evaluated emergent and recurring concepts from the participant's answers and further grouped them into several categories. The number of recurring concepts was counted to determine a specific concept's frequency or occurrence. All authors in this study discussed and decided the definition and grouping for each category. Considering the unique nature of the participant's answers, we did not use a specific software package for the qualitative analysis.

## Results

There were 132 current endoscopy trainees and graduates of endoscopy training, or 24.58% of The Indonesian Society for Gastrointestinal Endoscopy members, who participated in this study. The participants were internal medicine specialists and gastroenterology fellows, and subspecialists. The demographic characteristic of the participants is presented in Table 1.

**Table 1:** Characteristics of study participants

Characteristics	Frequency	Percentage
Age group		
<40 years	22	16.7
40–60 years	97	73.5
>60 years	13	9.8
Gender		
Male	93	70.5
Female	39	29.5
Year of endoscopy training		
<2000	5	3.8
2001–2010	28	21.2
2011–2020	86	65.2
2021–now	13	9.8
Regions of origin		
Aceh	6	4.5
Bali	4	3.0
Bangka Belitung	1	0.8
Banten	4	3.0
Bengkulu	1	0.8
Yogyakarta	5	3.8
Jakarta	15	11.4
Jambi	2	1.5
West Java	17	12.9
Central Java	18	13.6
East Java	17	12.9
West Kalimantan	3	2.3
South Kalimantan	1	0.8
East Kalimantan	1	0.8
Lampung	4	3.0
West Nusa Tenggara	5	3.8
Papua	1	0.8
Riau	5	3.8
South Sulawesi	4	3.0
Southeast Sulawesi	1	0.8
North Sulawesi	2	1.5
West Sumatera	2	1.5
South Sumatera	2	1.5
North Sumatera	11	8.3

We further surveyed methods of endoscopy training as experienced by the study participants. Endoscopy trainees in Indonesia might encounter different training methods based on their training location or time. The procedures taught and whether participants received learning materials or pretests before training are also presented in Table 2.

**Table 2:** Methods and endoscopy training experience in Indonesia

	Frequency	Percentage
Training method		
Direct practice with supervision	51	38.6
Observation + direct practice with supervision	48	36.4
Simulation + observation + direct practice with supervision	33	25.0
Procedures		
Esophagoduodenoscopy (EGD)	131	99.2
Colonoscopy	130	98.5
Hemostatic endoscopy	72	54.5
Polypectomy	76	57.6
Ligation	108	81.8
Whether participants received learning materials before training		
Yes	112	84.8%
No	20	15.2%
Pretest before training		
Yes	91	68.9%
No	41	31.1%

**Figure 1:**
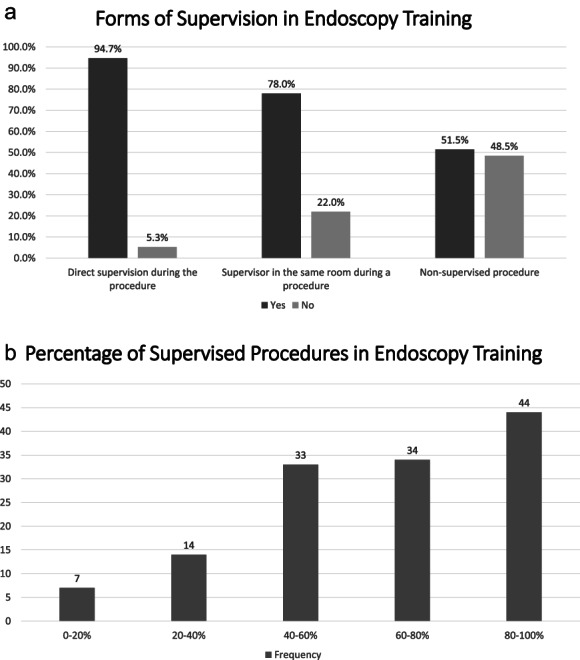
Supervision in endoscopy training: (a) Forms of supervision during endoscopy training. 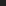, yes; 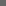, no. (b) Percentages of supervised procedures during endoscopy training. 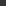, frequency.

Supervision is an essential part of medical training. From our survey, we found that all of the surveyed participants had received supervision during their endoscopy training. However, the degree or form of supervision differed in different participants. Figure 1 shows the type of supervision experienced during endoscopy training and the percentage of supervised procedures.

**Figure 2:**
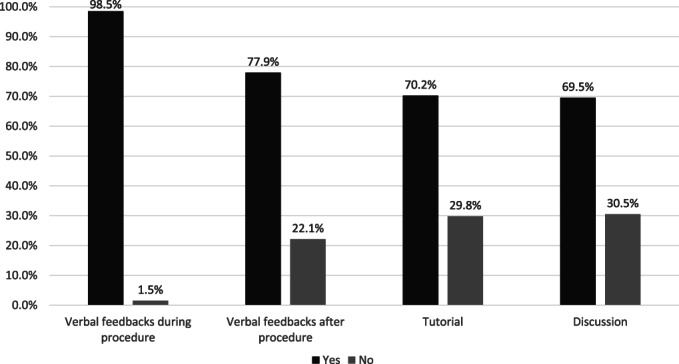
Forms of feedback in endoscopy training. 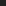, yes; 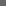, no.

During endoscopy training, trainers were encouraged to provide feedback to the trainees. Feedback might be delivered verbally during or after a procedure or through tutorials and discussions (Fig. 2). Of the 132 participants, 130 (98.5%) answered that they were given feedback during endoscopy training. Using a Likert scale to determine the importance of feedback for endoscopy trainees, 121 (91.7%) participants answered that feedbacks during endoscopy training were very important.

**Figure 3:**
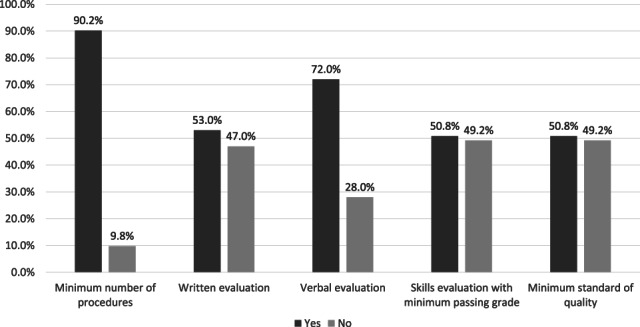
Assessment methods in endoscopy training. 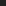, yes; 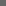, no.

Endoscopy trainees were expected to achieve a minimum competency after finishing their training. To ensure that every trainee achieves the required competency, a competence checklist and/or logbook might be given to monitor the endoscopy training. Of the 132 participants, 94 (71.2%) stated that they received a competence checklist during endoscopy training. Meanwhile, 109 (82.6%) participants used logbooks during the training. Endoscopic competency after training should be measured objectively with a formal assessment. However, only 93 (70.5%) participants answered that they were evaluated for each endoscopic skill, while only 82 (62.1%) had a formal assessment during their training. Several methods could be used to assess a trainee's competency. A training center might apply some or all of the methods of assessment. Figure 3 presents forms of assessment methods used in endoscopy training.

We also evaluated trainees' satisfaction with their current or past endoscopy training experience. One‐hundred and four (78.8%) participants stated that the current endoscopy training method is satisfactory. Meanwhile, 110 (83.3%) participants answered that the training materials had been delivered effectively. However, only 79 (59.8%) participants agreed that the current endoscopy training duration (3 months) had been enough to achieve the desired competence. The trainees' level of satisfaction on various aspects of endoscopy training is presented in Table 3.

**Table 3:** Trainee's level of satisfaction in endoscopy training

	Level of satisfaction, *n* (%)				
Aspects of endoscopy training	1	2	3	4	5
Training method	1 (0.8)	2 (1.5)	13 (9.8)	56 (42.4)	60 (45.5)
Competency achieved after training	1 (0.8)	3 (2.3)	28 (21.2)	52 (39.4)	48 (36.4)
Endoscopy trainers	1 (0.8)	2 (1.5)	17 (12.9)	35 (26.5)	77 (58.3)
Training environment	1 (0.8)	2 (1.5)	16 (12.1)	44 (33.3)	69 (52.3)
Training feedbacks	1 (0.8)	3 (2.3)	17 (12.9)	52 (39.4)	59 (44.7)
Assessment methods	1 (0.8)	4 (3.0)	21 (15.9)	46 (34.8)	60 (45.5)

1 represented most unsatisfactory; 5 represented most satisfactory.

**Figure 4:**
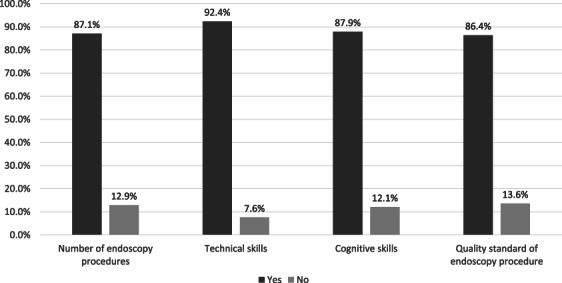
Important factors in endoscopy training (according to endoscopy trainees). 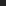, yes; 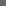, no.

Participants were asked about factors they deemed important in endoscopy training, including the number of procedures performed during training, technical skills, cognitive skills, and quality standard of endoscopy procedures (Fig. 4). Technical skills were defined as the ability to perform endoscopic maneuvers. Cognitive skills were defined as the knowledge of indications of procedures and pathology found during endoscopy. Quality of endoscopy procedures was defined as the duration of procedures, adenoma detection rate, and adverse effects or complications after procedures.

To complete the assessment regarding the trainee's satisfaction with endoscopy training, participants were asked to answer open‐ended questions regarding barriers to achieving competency in the current training method and their suggestions for future endoscopy training. We analyzed the participants' answers using content analysis and present the most found concepts and the representative examples in Tables 4 and 5. The representative examples were translated from the Indonesian language to English by the authors.

**Table 4:** Barriers to achieving competency in endoscopy training

Identified concepts	Frequency	Definition	Representative examples
Insufficient number of patients	25	The number of endoscopy patients or procedures is insufficient, so trainees cannot practice enough	“There were not many colonoscopy cases when I trained, so I felt not proficient enough to perform the procedure after the training” “I did the training during the early COVID‐19 pandemic, so there were fewer endoscopic patients”
Incomplete tools or endoscopy equipment	16	Damaged endoscopic tools or incomplete accessory equipment so that trainees cannot practice optimally or cannot perform certain procedures	“There was a shortage of accessory equipment, so we could not perform therapeutic endoscopic procedures” “Sometimes the endoscopic tool is damaged, so we could not practice”
Lack of variety of procedures	15	Lack of variety in procedures or cases was found during training	“The endoscopy cases were not varied enough”
Lack of guidance regarding difficult cases or procedures	10	Guidance or discussion related to difficult cases or procedures during training was lacking	“There was a lack of guidance regarding difficult cases and maneuvers and how to overcome them”
Limited time or opportunity to practice	7	Time or opportunity for independent practice is limited for trainees, so they feel less skilled or less confident	“We did not have enough time for supervised or independent practice” “We were not acquainted well enough with the endoscopic tools at the beginning, so we lacked confidence when we had to do the procedure on a patient”

**Table 5:** Suggestions for future endoscopy training

Identified concepts	Frequency	Definition	Representative examples
Increasing variety of cases or procedures	11	Increasing the variety of cases or types of procedures learned in endoscopy training	“There should be more types of cases and procedures learned during the training”
Standardization of endoscopy training	10	Standardization of endoscopic training held by various training centers by developing a national standard curriculum or manual	“There should be a standard national module or manual for endoscopy training” “There should be a comprehensive curriculum for endoscopy training”
Increasing accessibility to endoscopy training	10	Increasing access to training by increasing the number of endoscopy training centers or by increasing the number of times training is held in a year	“Endoscopy training should be held more often” “The access to training should be widened because the training center was too far, and I had to leave my job and my family to train”
Therapeutic endoscopic training	7	Adding therapeutic endoscopy as a part of endoscopy training	“Trainees should be given more opportunities to learn therapeutic or interventional endoscopy” “Simple therapeutic cases should be added to increase the trainee's skills”
Better supervision	7	Increasing direct supervision during training	“Participants must be given training under the direction of a supervisor so they can understand the technique in performing endoscopy better in accordance with the competencies they are expected to master” “Trainees should be supervised directly at the beginning, then slowly given the opportunity to practice independently”
Use of simulators	7	Using simulators to support endoscopy training	“I suggest training with simulators before performing procedures on a patient” “Simulators should be used to allow trainees to learn therapeutic endoscopy or rarer cases or procedures”

## Discussion

This study has presented the current state of GI endoscopy training in Indonesia and the barriers and suggestions to improve it according from the trainees' point of view. Almost all participants have performed esophagogastroduodenoscopy (EGD; 99.2%) and colonoscopy (98.5%) during their training, and a considerable percentage also performed some therapeutic endoscopy such as ligation (81.8%), polypectomy (57.6%), and hemostatic endoscopy (54.5%). As presented in Table 3, most participants were satisfied with the current endoscopy training (Likert scale 4–5).

All participants reported that they had been supervised during their endoscopy training. However, there are various degrees of endoscopy training supervision. In the beginning, the trainees will perform the procedure under direct supervision. As the training progresses, trainees will be expected to be able to perform the procedure without direct supervision. Although almost all (94.7%) trainees stated that they had been trained under direct supervision, only 51.5% reported that they had performed unsupervised procedures during their training, showing that currently there are varied degrees of supervision in different training centers or with different supervisors. Current endoscopy training guidelines from various countries suggest dividing the training into several phases. In the first phase, trainees are allowed to perform endoscopy procedures only under direct supervision. However, after formative evaluation using Direct Observation of Procedural Skills (DOPS), the trainees might be allowed to perform the procedures with a lesser degree of supervision (e.g. indirect supervision with the supervisor in the same unit) and later independently.^5^, ^6^ This type of supervision is also a proposed suggestion from the current and previous endoscopy trainees in Indonesia for future endoscopy training.

Feedback is an essential part of learning procedural skills. Previous studies on colonoscopy simulation training had shown that trainees who received feedback during their training had a better proficiency level outcome than those who did not receive any feedback.^7^, ^8^ Supervisors might give various forms of feedback during endoscopy training. Most participants (98.5%) in this study received concurrent verbal feedback during a procedure. However, some participants also received other forms of feedback, such as terminal feedback at the end of a procedure, tutorial, or discussion. While verbal feedback is given in all aspects of training, tutorial and discussion are usually reserved for more complex procedures or cases.

Different ways of giving feedback might provide different training results. A meta‐analysis by Hatala *et al*. found that terminal feedback might be more effective than concurrent feedback for skill retention in a novice learner. According to Cognitive Load Theory, concurrent feedback might induce information load, making it harder to understand the feedback. Another theory is that concurrent feedback might result in the over‐reliance of trainees on the feedback. However, more complex procedures might benefit more from concurrent feedback.^9^ In providing feedback, Dilly *et al*. suggested discussing the training session's goals with the trainee before a procedure, giving minimal feedback during the procedure, and providing the rest of the feedback later after the procedure. Trainees should be given the opportunity to assimilate the feedback and do a self‐reflection to set their goals for the next training session.^10^

There are several ways to assess a trainee's competence in endoscopy training, including a minimum number of procedures, verbal or written evaluation, skills evaluation, and a minimum standard of quality. The minimum number of procedures is the most common form of assessment (90.2%) among the trainees in Indonesia. A logbook or a competence checklist is usually used to record the procedures or cases the trainees have encountered. The current Joint Advisory Group (JAG) consensus for training and certification of EGD also recommends 250 procedures as the minimum before eligibility for summative assessment.^5^ Meanwhile, Ward *et al*. have calculated that the number of EGDs required to achieve a 95% completion rate in trainees was 187 procedures.^11^ However, a minimum number of procedures should not be the only form of assessment for competency. JAG also recommends that DOPS be used as the assessment tool for competency in EGD, and several formative DOPS should be performed before a trainee can undergo a summative assessment.

From our survey, only 62.1% of endoscopy trainees in Indonesia had a formal assessment during their training. Ensuring a trainee's competence or the uniformity of training results without a formal assessment is difficult. Therefore, a system including formal assessment and passing grade criteria should be established for future endoscopy training. Most of our participants agreed that the number of minimum procedures, technical skills, cognitive skills, and the minimum standard of quality are all important in endoscopy training. A training module or curriculum should incorporate these four factors in endoscopy training. The Korean Society of Gastrointestinal Endoscopy (KSGE), for example, have included basic knowledge and disease contents, basic procedures, and a minimum number of procedures as necessary to achieve competency for each procedure in their training guidelines.^12^

Most survey participants were satisfied with their endoscopy training (Likert scale 4–5). However, there are still barriers to achieving competency, as suggested by the trainees. A national curriculum or module should be established as a guideline for endoscopy training. Currently, a competency‐based system is more recommended than a time‐based system to ensure that all trainees are competent to perform endoscopy procedures safely and effectively after training. Competence is defined as the minimum level of skill, knowledge, and/or expertise derived through training and experience required to safely and proficiently perform a procedure.^13^ A formal assessment system should also be established to determine the trainees' competence.

There are also several other barriers in endoscopy training that might be unique to Indonesia as a developing country. The number of endoscopy patients, which equals the number of endoscopy procedures for the trainees, is still considered too low, and the variety of endoscopy cases is still lacking. This might be due to the low number of referrals from primary health services to secondary or tertiary health centers which provide endoscopy services. In recent years, the COVID‐19 pandemic also affected the number of endoscopy patients. To overcome it, simulators could be used to provide more opportunities for trainees to practice independently. In settings with low numbers of patients or cases, the use of simulators could provide the chance for trainees to practice without relying on the number of patients who will undergo endoscopy procedures. Moreover, different types of simulators could be developed to provide more variety of cases. However, we still consider it important for trainees to encounter more varieties of cases in real patients, and simulators should be used only as an additional training method instead of replacing real cases completely. Incorporating simulators in an endoscopy training module is also beneficial at the early phase of training as both a learning and an assessment tool before trainees are allowed to perform the procedure on a patient. Simulation‐based mastery learning (SBML) is currently one of the proposed methods for procedural task training, as it combines a competency‐based curriculum with simulation‐based training.^14^

Some endoscopy centers in Indonesia still have incomplete endoscopic tools and equipment, preventing them from performing complex procedures, such as interventional or therapeutic endoscopy. Some trainees also find the training centers not accessible enough, since the trainings are usually held only in big or capital cities far from their own hospitals or workplaces. A formal curriculum for endoscopy training should also include the necessary endoscopic tools and equipment for a training center, which could be a foundation for the centers to propose more budget for their endoscopy training.

## Conclusion

This study was conducted to assess the current state of endoscopy training, the barriers to achieving competency, and suggestions for future endoscopy training in Indonesia from the trainees' point of view. Although most participants were found to be satisfied with the training methods and their competency after training, several things can still be improved for a better endoscopy training environment and result in Indonesia. We suggest designing a national curriculum for endoscopy training and a formal assessment system to ensure the trainees' competence, which will further increase the quality of endoscopic patient care in Indonesia.

## Supporting information


**Appendix S1.** Supporting information.
