# Supplementary material for: Needs assessment of basic gastrointestinal endoscopy training: A qualitative study in Indonesia
Source: JGH Open. 2023 Nov 20;7(12):928–35. doi: 10.1002/jgh3.13004 (PMC10757483; doi:10.1002/jgh3.13004)
Supplement: Supplementary file 1 — Appendix S1. Supporting information. [file JGH3-7-928-s001.docx]

**Manuscript title: Need Assessment of Basic Gastrointestinal Endoscopy Training: A Qualitative Study in Indonesia**

**Supporting information**

**Appendix 1 – Questionnaire for Endoscopy Training Graduates**

1. Full name:
2. Age:
3. Gender: Male/Female
4. Last education:
5. Current workplace:
6. In what year did you join basic endoscopy training?
7. What kind of training method did you experience? (You may choose more than one answer)
   1. Observation
   2. Performing procedures with supervision
   3. Simulation/Simulator
8. What type of endoscopic procedures have you performed? (You may choose more than one answer)
   1. EGD
   2. Colonoscopy
   3. Hemostatic endoscopy
   4. Polypectomy
   5. Ligation
9. How many endoscopic procedures have you performed until now? (please mention the procedure name and the amount, e.g., EGD 10 times, colonoscopy 20 times, etc.)
10. Did you receive learning materials about endoscopy before the training?
    1. Yes
    2. No
11. Did you have a pretest before training to measure your previous knowledge about endoscopy?
    1. Yes
    2. No
12. Did you receive supervision from the trainers during the endoscopy training?
    1. Yes
    2. No
13. What kind of supervision did you receive during the training? (You may choose more than one answer)
    1. Direct supervision during the procedure
    2. Supervisor in the same room during a procedure
    3. Non-supervised procedures
14. How many percentages of endoscopic procedures were supervised during the training?
    1. 0 – 20%
    2. 20 – 40%
    3. 40 – 60%
    4. 60 – 80%
    5. 80 – 100%
15. Did you get feedback from the trainers/supervisors during the training?
    1. Yes
    2. No
16. What kind of feedback did you receive from the trainers/supervisors? (You may choose more than one answer)
    1. Verbal feedback during a procedure
    2. Verbal feedback after a procedure
    3. Tutorial
    4. Discussion
17. In your opinion, how important is the feedback given by the trainers/supervisors? (Choose a number from 1 to 5 where 1 is very insignificant and 5 is very significant)
18. Did you receive a competency checklist of endoscopic procedures during the training?
    1. Yes
    2. No
19. Did you need to fill out a logbook for endoscopic procedures during the training?
    1. Yes
    2. No
20. Do you think access or opportunity to practice to achieve endoscopic competency was enough during the training?
    1. Yes
    2. No
21. Were there assessments for each endoscopic skill?
    1. Yes
    2. No
22. Was there a formal assessment system during the training to test your skill in performing endoscopy procedures?
    1. Yes
    2. No
23. Which type of assessment did you experience during the endoscopy training? (You may choose more than one answer)
    1. Minimum number of procedures
    2. Written evaluation
    3. Verbal evaluation
    4. Skills evaluation (with a simulator or on a patient) with a minimum passing grade
    5. Minimum standard of quality
24. Did you feel proficient in performing endoscopy with the training method provided?
    1. Yes
    2. No
25. Have all materials been delivered effectively during the training?
    1. Yes
    2. No
26. Was the endoscopic training time sufficient to master all endoscopic skills?
    1. Yes
    2. No
27. What obstacles or difficulties did you experience during the endoscopy training?
28. In your opinion, what endoscopic actions or skills were still not optimal in endoscopic training?

For questions 29 – 34, participants were asked to choose from numbers 1 - 5 (1=dissatisfied;2=not satisfied;3=quite satisfied;4=satisfied 5=very satisfied)

1. In general, how satisfied were you with the endoscopic training methods provided?
2. Were you satisfied with your competency after/during the endoscopy training?
3. How satisfied were you with the endoscopic training instructors?
4. How satisfied were you with the environment from the endoscopy training?
5. How satisfied were you with the feedback you received during the endoscopy training?
6. How satisfied are you with the assessments carried out in the endoscopy training?
7. In your opinion, what are the important factors to consider in endoscopy training? (You may choose more than one answer)
   1. The number of procedures performed
   2. Cognitive skills (knowledge of endoscopy indications, pathology, maneuvers)
   3. Technical skills (skills in performing endoscopic maneuvers)
   4. Standard quality of endoscopy results (endoscopic procedure duration, adenoma detection rate, side effect rate)
8. Do you have any suggestions to improve the current endoscopy training?
